# Comparative study on functional outcomes of bipolar hemiarthroplasty versus total hip arthroplasty in elderly patients with displaced femoral neck fractures

**DOI:** 10.12669/pjms.41.9.11781

**Published:** 2025-09

**Authors:** Zhenning Liu, Jun Li, Daojian Zhang, Hao Wu

**Affiliations:** 1 Zhenning Liu Department of Orthopedics, Peking University First Hospital, Beijing 100034, China; 2 Jun Li Department of Orthopedics, Peking University First Hospital, Beijing 100034, China; 3 Daojian Zhang Department of Orthopedics, Peking University First Hospital, Beijing 100034, China; 4 Hao Wu Department of Orthopedics, Peking University First Hospital, Beijing 100034, China

**Keywords:** Bipolar hemiarthroplasty, Total hip arthroplasty, Elderly, Displacement, Femoral neck fracture

## Abstract

**Objective:**

To compare the functional outcomes of bipolar hemiarthroplasty (BHA) versus total hip arthroplasty (THA) in elderly patients with displaced femoral neck fractures (DFNFs).

**Methodology:**

This was a retrospective comparative study. Ninety-two elderly patients with DFNFs who were treated at Peking University First Hospital from January 2022 to October 2023 were enrolled in the study. The participants were randomly assigned into an observation group (BHA, *n=* 46) and a control group(THA, *n=*46) using a random number table. Perioperative parameters were compared and complications were analyzed between the two groups. Postoperative pain was rated using the Visual Analog scale (VAS) at 12, 24, 48 and 72 hours and seven days postoperative. The Harris Hip Score (HHS) was recorded at one month(T_1_), three months(T_2_), six months(T_3_) and one year(T_4_) postoperative.

**Results:**

The observation group significantly outperformed the control group in duration of operation (DoO), intraoperative blood loss (IBL), postoperative drainage volume (PDV), length of hospital stay (LoHS), incision length (IL) and ambulation time (AT) (*P<*0.05, respectively). The two groups exhibited significant time, group and group-by-time interaction effects for both the VAS and HHS (*P<* 0.05, respectively). The observation group had lower VAS scores at 12, 24, 48 and 72 hours post-surgery and higher HHS at T_1_ and T_2_ compared with the control group (*P <* 0.05, respectively).

**Conclusion:**

For elderly patients with DFNFs, BHA, compared with THA, offers shorter DoO, less surgical trauma and better early functional recovery, facilitating postoperative rehabilitation while reducing surgical risks.

## INTRODUCTION

Femoral neck fractures are a common type of hip fracture among the elderly, with an increasing incidence due to population aging.^1^,^2^ These fractures, often caused by falls or impacts, not only impair limb function and reduce the quality of life but can also pose life-threatening risks to older adults if not treated promptly.^3^,^4^ The Garden classification system is widely used in clinical practice to categorize femoral neck fractures based on the degree of displacement, dividing them into four types: Type-III and Type-IV fractures are displaced fractures associated with more severe clinical symptoms.^5^,^6^ Displaced femoral neck fractures (DFNFs) have a significant impact on the blood supply to the femoral head, making hip arthroplasty the primary treatment modality in current clinical practice.^4^,^7^,^8^

However, the choice between total hip arthroplasty (THA) and hemiarthroplasty (HA) remains a subject of debate, as both surgical approaches have distinct advantages and drawbacks.^7^-^9^ Reportedly, THA is associated with better functional outcomes but carries a higher risk of postoperative dislocation and increased surgical trauma due to acetabular replacement. In contrast, HA involves less surgical trauma but may lead to long-term acetabular wear, potentially requiring revision surgery. There are two types of HA prostheses: unipolar and bipolar. Bipolar hemiarthroplasty (BHA) prostheses, featuring two articulating surfaces in the head design, are advantageous in reducing acetabular cartilage wear.^4^ However, it remains unclear whether BHA or THA can produce better functional outcomes. This study included 92 elderly patients with DFNFs, aiming to compare the effects of BHA and THA on postoperative functional recovery.

## METHODOLOGY

This was a retrospective comparative study. Ninety-two elderly patients with DFNFs who were treated at Peking University First Hospital between January 2022 to October 2023. Using a random number table, the patients were divided into an observation group (*n* = 46) and a control group (*n* = 46).

### Ethical Approval:

The study was approved by the Institutional Ethics Committee of Peking University First Hospital (No.: 2021[484-001]; Date: March 28, 2022) and written informed consent was obtained from all participants.

### Inclusion criteria:

- Fresh fractures with an injury-to-surgery time of less than two weeks.
- Garden Type-III-IV fractures.
- Aged ≥60 years.
- Normal cognitive function.

### Exclusion criteria:

- Previous hip arthroplasty.
- Concurrent hip conditions such as femoral head necrosis or osteoarthritis.
- Malignant tumors.
- Femoral deformities.
- Neuromuscular conditions such as myasthenia gravis or Parkinson’s disease.

All procedures were performed under general anesthesia or spinal anesthesia. Antibiotics were administered intravenously 30 min before surgery for infection prophylaxis. Patients were positioned in the lateral decubitus position on the healthy side. A posterior-lateral approach to the hip joint was used, entering along the posterior edge of the gluteus Medius. The short external rotators were cut and the posterior joint capsule was incised to expose the hip joint. The femoral neck was sawed off and the femoral head was removed.

**Fig.1:**
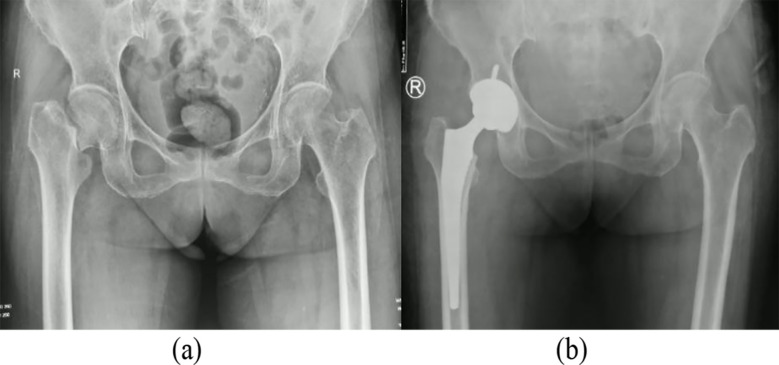
Preoperative and postoperative X-ray images of THA. ***Note:*** Female, 68 years old, underwent THA for a right femoral neck fracture. (a) Preoperative X-ray; (b) Postoperative X-ray.

**Fig.2:**
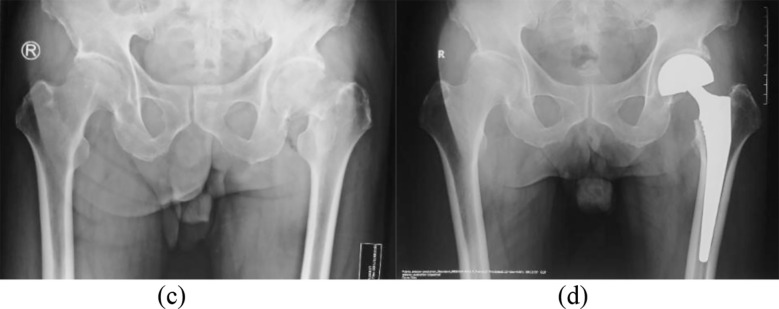
Preoperative and postoperative X-ray images BHA. ***Note:*** Male, 70 years old, underwent BHA for a left femoral neck fracture. (c) Preoperative X-ray; (d) Postoperative X-ray.

In the control group, standard THA was performed. A biological acetabular prosthesis and a femoral prosthesis were press-fit into place. A femoral head prosthesis of appropriate size and neck length was selected. Post-reduction, joint stability was confirmed (Fig.1). In the observation group, BHA was performed using standard procedures. A biologic femoral prosthesis was press-fit and an appropriately sized bipolar femoral head prosthesis was selected based on the original femoral head size. Post-reduction, joint stability was confirmed (Fig.2).

For all cases, the posterior joint capsule was sutured and the short external rotators were reconstructed posterior to the greater trochanter. Drainage tubes were placed. Postoperatively, pain management and deep vein thrombosis (DVT) prevention measures were provided.

### Outcome Measures:

### Perioperative Parameters:

The following parameters were recorded for both groups: duration of operation (DoO), intraoperative blood loss (IBL), postoperative drainage volume (PDV), length of hospital stays (LoHS), incision length (IL), ambulation time (AT).

### Postoperative Pain Levels:

Postoperative pain was assessed at 12 h, 24 h, 48 h and 72 hours, as well as seven days, using the visual analog scale (VAS). The VAS score ranges from 0 to 10, with higher scores indicating more severe pain.

### Hip Joint Function:

Hip function was evaluated using the Harris Hip Score (HHS) at one month (T_1_), three months (T_2_), six months (T_3_) and one year (T_4_) postoperatively. The HHS assesses four components, namely function (47 points), pain (44 points), range of motion (ROM, 5 points) and deformity (4 points). The total score is 100, with higher scores indicating better recovery of hip function.

### Complications:

The incidence of the following complications within six months postoperative was recorded for both groups: incisional infection, pulmonary infection, urinary tract infection, DVT of the lower extremities and prosthesis dislocation. The follow-up work of all patients was completed by the same group of surgeons.

### Statistical analysis:

Data were analyzed using SPSS version 22.0. Measurement data were expressed as mean ± standard deviation (*X̅*±*S*), while categorical data were expressed as frequency and percentage (*n* [%]). Comparisons between groups were conducted using the t-test or chi-square (*χ*²) test, Repeated measures analysis of variance was employed to assess overall group effects. A *P*-value of <0.05 was considered statistically significant.

## RESULTS

**Fig.3:**
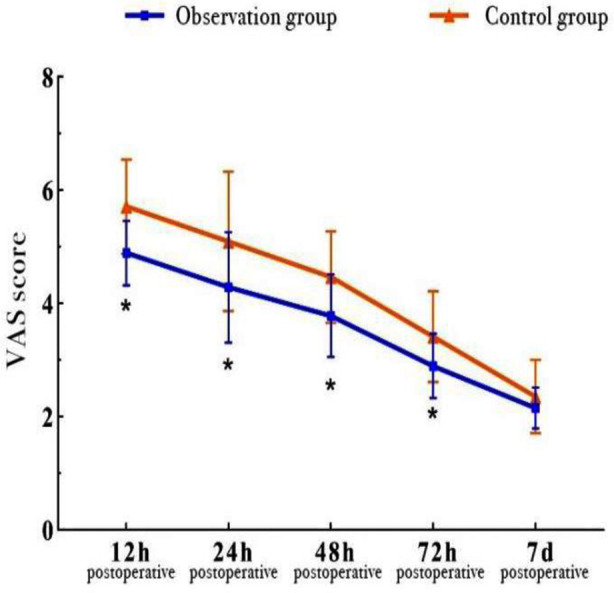
Comparison of postoperative pain levels between the two groups at different time points. ***Note:*** *P < 0.05 as compared with the control group.

The general characteristics of the two groups were comparable, with no statistically significant differences (all P > 0.05) Table-I. Compared with the control group, the observation group exhibited significantly shorter DoO, reduced IBL, lower PDV, shorter LoHS, smaller IL and earlier ambulation (P < 0.05, respectively) Table-II. Compared with the control group, the observation group reported significantly lower VAS scores at 12, 24, 48 and 72 hours postoperative (*P <* 0.05, respectively). No significant difference was observed in VAS scores between the two groups at seven days postoperative (Table-II and Fig.3). Significant differences were found in VAS scores over time, between groups and in group-by-time interactions (*P <* 0.05, respectively) Table-III.

**Table-I:** Comparison of baseline characteristics between the two groups.

Group	Sex, n(%)		Age (years)	BMI (kg/m²)	Cause of fracture, n(%)			Hypertension, n(%)	Diabetes, n(%)	Side of fracture, n(%)	
	Male	Female			Fall	High fall	Traffic accident			Left	Right
Observation group (*n =* 46)	15(32.61)	31(67.39)	70.28±2.95	24.50±1.76	33(71.74)	5(10.87)	8(17.39)	21(45.65)	15(32.61)	25(54.35)	21(45.65)
Control group (*n =* 46)	13(28.26)	33(71.74)	69.96±3.03	24.48±1.66	32(69.56)	4(8.70)	10(21.74)	18(39.13)	16(34.78)	23(50.00)	23(50.00)
*χ²/t*-value	0.205		0.523	0.061	0.349			0.401	0.049	0.174	
*P*-value	0.650		0.602	0.952	0.840			0.527	0.825	0.676	

**Table-II:** Comparison of perioperative parameters between the two groups.

Group	n	DoO (min)	IBL (mL)	PDV (mL)	LoHS (d)	IL (cm)	AT (d)
Observation group	46	76.26±8.34	181.59±28.14	125.17±37.54	8.30±2.82	10.24±1.45	2.44±0.50
Control group	46	92.46±10.04	329.07±34.14	205.74±45.37	10.74±4.47	13.04±2.23	3.00±0.47
*t-value*		8.415	22.609	9.280	3.131	7.139	5.571
*P-value*		<0.001	<0.001	<0.001	0.002	<0.001	< 0.001

**Table-III:** Comparison of postoperative pain levels between the two groups.

Group	VAS score				
	12 h postoperative	24 h postoperative	48 h postoperative	72 hours postoperative	7 d postoperative
Observation group (*n =* 46)	4.89±0.57	4.28±0.98	3.78±0.73	2.89±0.57	2.15±0.36
Control group (*n =* 46)	5.71±0.83	5.09±1.23	4.46±0.81	3.41±0.80	2.35±0.65
*t*-value	5.731	3.485	4.308	3.659	1.733
*P*-value	<0.001	0.001	<0.001	<0.001	0.086
*F*-value	F_time_ = 106.029, F_group_ = 1.867, F_group-by-time_ = 3.758				
*P*-value	P_time_ < 0.001, P_group_ = 0.022, P_group-by-time_ = 0.007				

At T_1_ and T_2_, the HHS values of the observation group were significantly higher than those of the control group (*P <* 0.05, respectively). At T_3_ and T_4_, there was no significant difference between the two groups. Significant differences were observed in the HHS over time, between groups and in group-by-time interactions (*P <* 0.05, respectively) Table-IV. Within six months postoperative, the complication rate in the observation group was lower than in the control group. However, the difference was not statistically significant (*P >* 0.05) Table-V.

**Table-IV:** Comparison of the HHS between the two groups.

Group	HHS			
	T_1_	T_2_	T_3_	T_4_
Observation group (n = 46)	86.87±5.69	90.65±7.23	93.22±2.57	90.04±3.64
Control group (n = 46)	73.61±5.89	85.15±8.13	92.96±2.05	89.11±3.28
t-value	9.326	3.428	0.537	1.294
P-value	< 0.001	< 0.001	0.592	0.199
F-value	F_time_ = 220.691, F_group_ = 35.662, F_group-by-time_ = 34.970			
P-value	P_time_ < 0.001, P_group_ < 0.001, P_group-by-time_ < 0.001			

***Note:*** T_1_ = 1 month postoperative, T_2_ = 3 months postoperative, T_3_ = 6 months postoperative, T_4_ = 1 year postoperative.

**Table-V:** Comparison of complication rates between the two groups, n(%).

Group	Incision infection	Pulmonary infection	Urinary tract infection	Lower limb DVT	Prosthesis dislocation	Overall incidence
Observation group (*n =* 46)	0	0	0	1(2.17)	0	1(2.17)
Control group (*n =* 46)	0	1(2.17)	1(2.17)	2(4.35)	1(2.17)	5(10.87)
*χ²*-value	-	-	-	-	-	2.853
*P*-value	-	-	-	-	-	0.091

## DISCUSSION

A comparative study was conducted on BHA and THA, two frequently used hip replacement procedures in clinical practice. Our study results showed that the BHA group had shorter DoO, less IBL, lower PDV, shorter LoHS, smaller IL and earlier ambulation compared with the THA group. Similar findings have been reported by other researchers, indicating that THA requires longer DoO and causes more IBL, as the acetabular operation involved in THA contributes to prolonged operation and greater surgical trauma.^10^-^13^ On the other hand, our postoperative pain assessment revealed that within the first 72 hours postoperative, the BHA group experienced relatively lower pain levels. This might be explained by the relatively minor surgical trauma associated with HA (shorter DoO, smaller IL and no need for acetabular replacement), resulting in less pain during the first three postoperative days. By the end of the first postoperative week, there was no significant difference in pain levels between the two groups.

This underscores the importance of restoring hip joint function, alleviating lower limb mobility restrictions and reducing pain in the clinical treatment for elderly patients with DFNFs. DFNFs greatly impact the blood supply to the femoral head. Internal fixation procedures for these fractures are associated with a high failure rate and suboptimal functional outcomes. Consequently, hip replacement surgery has become the preferred clinical treatment in recent years.^3^,^14^ Regarding functional outcomes, some studies support that THA yields superior results compared with HA^11^,^15^-^17^, while others reported functional outcomes of the two procedures are similar.^8^,^18^,^19^ However, very few studies have focused on comparing the early functional recovery following these two surgical approaches. In this study, through a one-year follow-up of all patients, we found that the HHS at one and three months postoperatively were higher in the BHA group compared with the THA group. This may be due to the relatively smaller surgical trauma associated with BHA, earlier ambulation and less pain during the early postoperative period, which facilitated earlier out-of-bed activities and functional exercises, promoting early functional recovery.

However, at six months and one year postoperative, there was no significant difference in the functional scores between the two groups, indicating similar long-term functional outcomes. Additionally, our study found that the incidence of postoperative complications was lower in the BHA group compared with the THA group. The BHA group had only one case of lower limb DVT, while the THA group had one case each of pulmonary infection, urinary tract infection and prosthesis dislocation, as well as two cases of lower limb DVT. However, these differences showed no statistical significance. Several comparative studies and meta-analyses have already confirmed that the dislocation rate is higher in THA than in HA.^8^,^11^-^12^ However, Lewis et al.^12^ pointed out in their meta-analysis that postoperative dislocation tends to occur later in HA receivers than in those with THA and the incidence after a follow-up period of over four years showed no significant difference.

In a large-scale data analysis conducted by Edelstein et al.^11^, a retrospective study was performed on 61,695 elderly patients aged over 65 with DFDFs. Of these, 10,268 patients (16.6%) underwent THA and 51,427 (83.4%) underwent HA. Follow-up at 12 months postoperative confirmed that the dislocation risk was significantly higher in the THA group than in the HA group, but the overall dislocation rates in both groups remained low (2.9% in the THA group vs. 1.9% in the HA group). The authors suggested that this difference might not be clinically significant.

In our study, there was no statistically significant difference in the incidence of postoperative complications between the two groups. However, the HA group had fewer postoperative complications, probably because of the smaller surgical trauma, less IBL and earlier ambulation. These factors can effectively reduce the physical impact of surgery, potentially lowering the risk of developing complications. A concern regarding HA is the potential wear of the acetabular cartilage caused by the prosthesis, which may lead to pain and require revision surgery.^9^,^17^

Given the one-year follow-up in this study, post-HA acetabular cartilage wear was not assessed. A multicenter randomized controlled trial that included 1,495 patients showed no difference in revision rates between THA and HA for the treatment of DFDFs at two year postoperative.^8^ The retrospective analysis by Yeung et al.^20^ studied 4,516 patients who underwent HA for DFNFs between 1998 and 2017, finding that 99 patients required revision surgery for various reasons, with acetabular wear accounting for a revision rate of merely 0.66%. The authors concluded that HA remains a favorable treatment option for DFNFs.

### Limitations:

However, the limitation of this study is a small number of samples. In view of this, more samples should be included in future studies to further validate the findings of this study.

## CONCLUSIONS

Based on the above studies, compared with THA, BHA for treating elderly patients with DFNFs offers shorter DoO, less surgical trauma and better early functional recovery, which may benefit postoperative recovery and reduce surgical risks. However, the long-term outcomes of these two procedures still require further follow-up and observation.

### Authors’ Contributions:

**ZL** and **JL:** Conceived and designed the study, included the patients. Contributed to drafting and revising of article, critical appraisal of findings with literature, final approval and responsibility for integrity of work.

**DZ** and **HW:** Performed the statistical analysis, critical review and participated in its design.

All authors read and approved the final manuscript.
